# Aptamer-based technologies for extracellular vesicle analysis and engineering: from isolation to functional applications

**DOI:** 10.20517/evcna.2026.106

**Published:** 2026-09-18

**Authors:** Xuefei Li, Youyu Zhang, Danping Zhang, Theerakamol Pengsakul, Ying Qiao, Jiang Zheng, Lixing Huang

**Affiliations:** ^1^State Key Laboratory of Mariculture Breeding, Fisheries College, Jimei University, Xiamen 361021, Fujian, China.; ^2^Institute of Electromagnetics and Acoustics and Key Laboratory of Electromagnetic Wave Science and Detection Technology, Xiamen University, Xiamen 361005, Fujian, China.; ^3^Health and Environmental Research Center (HERC), Faculty of Environmental Management, Prince of Songkla University, Hat Yai 90110, Thailand.; ^4^Fourth Institute of Oceanography, Ministry of Natural Resources, Beihai 536000, Guangxi, China.; ^#^These authors contributed equally to this work and share first authorship.

**Keywords:** Extracellular vesicles, aptamers, SELEX, disease diagnosis, immunomodulation, drug delivery

## Abstract

Extracellular vesicles (EVs) are natural mediators of intercellular communication and hold promise for disease diagnosis and drug delivery, yet their intrinsic heterogeneity complicates isolation, molecular profiling, and targeted delivery. Aptamers, owing to their high affinity, specificity, chemical tunability, and scalable synthesis, offer a flexible molecular toolkit to address these challenges. In this review, we highlight recent progress in integrating aptamers into EV research across three interconnected domains: (1) the selection of aptamers against EV-associated surface markers; (2) the development of aptamer-based methods for EV capture and isolation; (3) the use of aptamer-functionalized EVs for targeted drug delivery and immunomodulation. We also discuss outstanding challenges, particularly the need for selection strategies that better preserve native EV epitope conformations and the rational design of multifunctional aptamer architectures. Looking forward, the convergence of aptamer technology with EV biology is expected to enable aptamer-drug conjugates and minimally invasive, dynamic liquid biopsy approaches for major diseases such as cancer, ultimately advancing early diagnosis and precision medicine.

## INTRODUCTION

Extracellular vesicles (EVs) are nanoscale lipid bilayer vesicles released by virtually all cell types into biofluids including blood, urine, and saliva^[1]^ (Figure 1, left and right panels: key structural components and biomarkers of exosomes, and their biogenesis via the endosomal system and release pathways). Originally dismissed as cellular debris, EVs are now recognized as key mediators of intercellular communication, shuttling proteins, nucleic acids, and lipids to recipient cells and thereby influencing diverse physiological and pathological processes^[2-4]^. However, the inherent heterogeneity of EVs, in terms of size, surface marker expression^[5]^, and molecular cargo, poses substantial challenges for their isolation, accurate molecular profiling, and targeted delivery. Conventional isolation methods, including ultracentrifugation, density gradient centrifugation, and size-exclusion chromatography, suffer from low purity, poor reproducibility, and compromised vesicle integrity^[6,7]^. These limitations underscore the urgent need for affinity-based strategies that enable specific and efficient EV capture.

**Figure 1:**
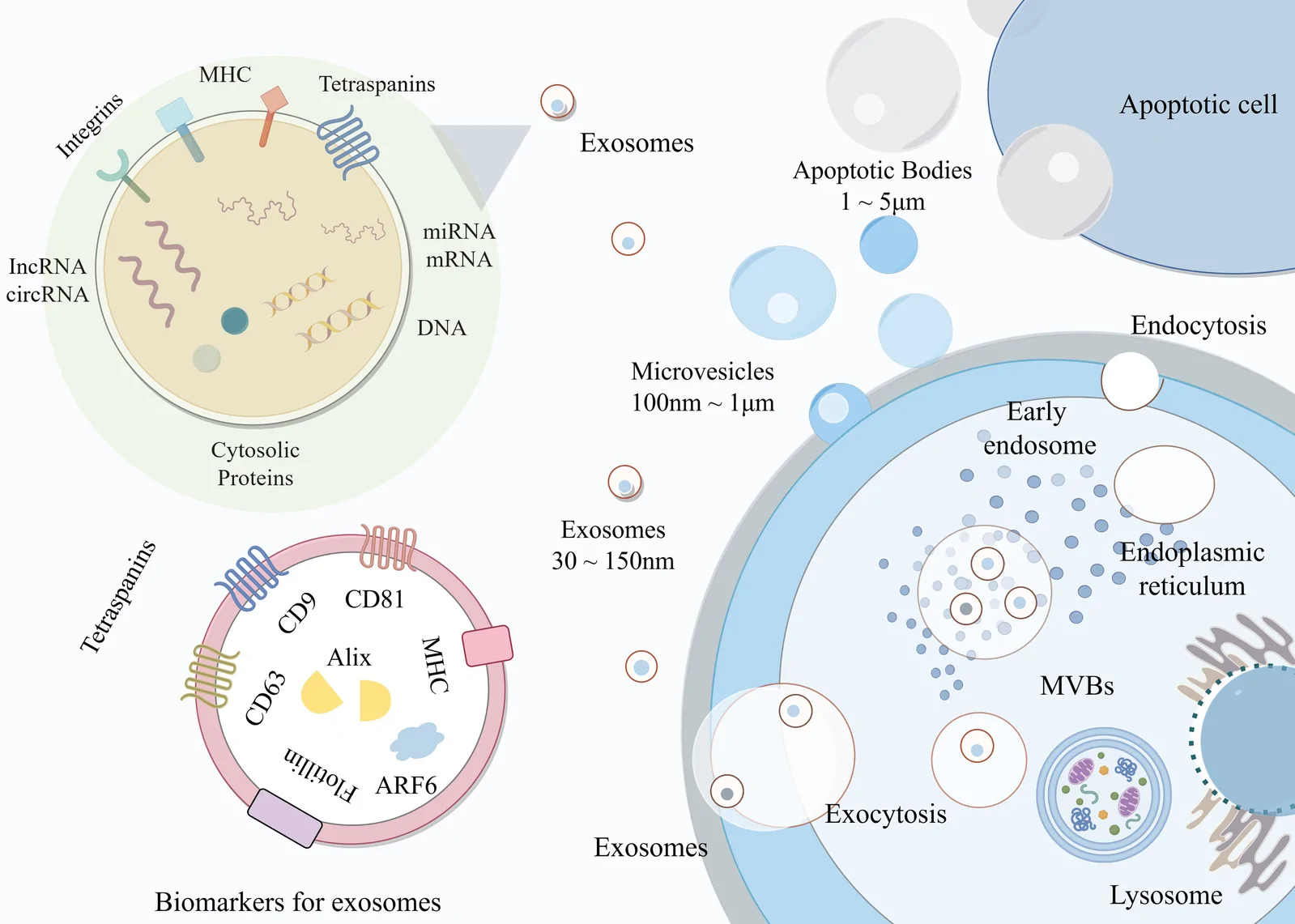
Schematic illustration of EV biogenesis, composition, and secretion. This schematic illustrates the heterogeneity and biogenesis of EVs, which are classified by size into apoptotic bodies, microvesicles, and exosomes. The left panels detail the structural composition of exosomes, highlighting various cargoes. The right panel maps the cellular biogenesis pathway, where endocytosis leads to the formation of early endosomes and subsequent MVBs. MVBs either fuse with the plasma membrane to release exosomes via exocytosis or are directed toward lysosomes for degradation. This figure was drawn with Figdraw and is an original work of the authors. Figdraw ID: WSWOUbbd28. EV: Extracellular vesicle; circRNA: circular RNA; lncRNA: long non-coding RNA; MHC: major histocompatibility complex; miRNA: microRNA; mRNA: messenger RNA; MVB: multivesicular body.

Aptamers, short, single-stranded DNA or RNA molecules generated through systematic evolution of ligands by exponential enrichment (SELEX), have emerged as compelling alternatives to antibodies in EV research^[3,8]^. Unlike antibodies, aptamers are chemically synthesized, offering batch-to-batch consistency, low immunogenicity, and facile chemical modification with fluorophores, drugs, or anchoring moieties^[9-13]^. More importantly, aptamers offer three distinct advantages that are particularly relevant to EV studies. First, EV SELEX enables the discovery of novel, disease-specific surface fingerprints without prior knowledge of EV surface epitopes, thereby overcoming the limitation of antibody-based approaches that target only predefined immunogenic proteins^[14,15]^. Second, aptamer-based EV capture can be reversed under mild conditions using complementary strand displacement, preserving EV integrity and cargo for downstream multi-omics analyses, a unique advantage over the harsh elution conditions (e.g., low pH, high salt, or organic solvents) required for antibody-based capture^[16,17]^. Third, the scalable, animal-free synthesis of aptamers eliminates the batch variability that has long plagued antibody-based EV diagnostics^[11]^. A comprehensive comparison of aptamers and antibodies is provided in Supplementary Table 1.

In this review, we examine recent advances in integrating aptamers into EV research across three interconnected domains: the selection of aptamers targeting EV surface markers; the engineering of aptamer-functionalized EVs for targeted drug delivery and immunomodulation; and the development of aptamer-based platforms for EV isolation and purification. We critically assess current challenges, in particular the need for selection strategies that preserve native EV epitope conformations and for the rational design of multifunctional aptamer architectures^[18]^. Finally, we offer perspectives on how aptamer-EV technologies could enable minimally invasive liquid biopsy strategies and accelerate the clinical translation of EV-based diagnostics and therapeutics.

## APTAMER SELECTION STRATEGIES: FROM SINGLE TARGETS TO INTACT EVs

### Principles of aptamer selection: single-marker versus whole-EV recognition

Aptamer-based recognition of EVs relies on specific three-dimensional interactions between aptamers and surface molecular features, including proteins, glycans, and lipid moieties^[19,20]^. Depending on the target form, two complementary strategies have emerged.

The first strategy targets predefined EV surface markers, such as tetraspanins (CD9, CD63, CD81) or disease-associated antigens^[17-21]^. Aptamers generated against these known epitopes offer well-defined binding sites and facilitate standardized assay development. For instance, slow off-rate modified aptamers (SOMAmers)®-based affinity proteomics has enabled simultaneous profiling of over 1,000 proteins in prostate cancer-derived EVs^[22]^, while artificial intelligence (AI)-assisted SELEX workflows reduced the number of laboratory enrichment cycles from conventional 12-15 to 5-7, as reported in a pooled analysis of 34 AI-integrated SELEX studies^[14]^. However, single-marker approaches are inherently limited by EV heterogeneity: even EVs from the same parental cell exhibit substantial variations in marker expression, and a single biomarker rarely captures full disease complexity^[23,24]^.

The second, more recent strategy employs intact EVs as selection targets^[25,26]^. Through positive selection against target EVs and counter-selection against controls, this approach yields aptamers that recognize combinatorial surface fingerprints, encompassing proteins, glycans, and lipids, without requiring prior knowledge of individual epitopes^[27]^. Such holistic recognition more faithfully reflects the physiological or pathological state of the EV-producing cells. Zhao and colleagues demonstrated this concept by developing a capillary electrophoresis-based, three-step SELEX against intact natural killer cell-derived EVs, obtaining aptamers with a Kd of 27.6 nM within five rounds^[28]^. Collectively, aptamers thus provide dual functionality, serving as antibody-like probes for known markers or as discovery tools to uncover unknown EV surface features. A schematic representation of the conventional SELEX workflow is shown in Figure 2.

**Figure 2:**
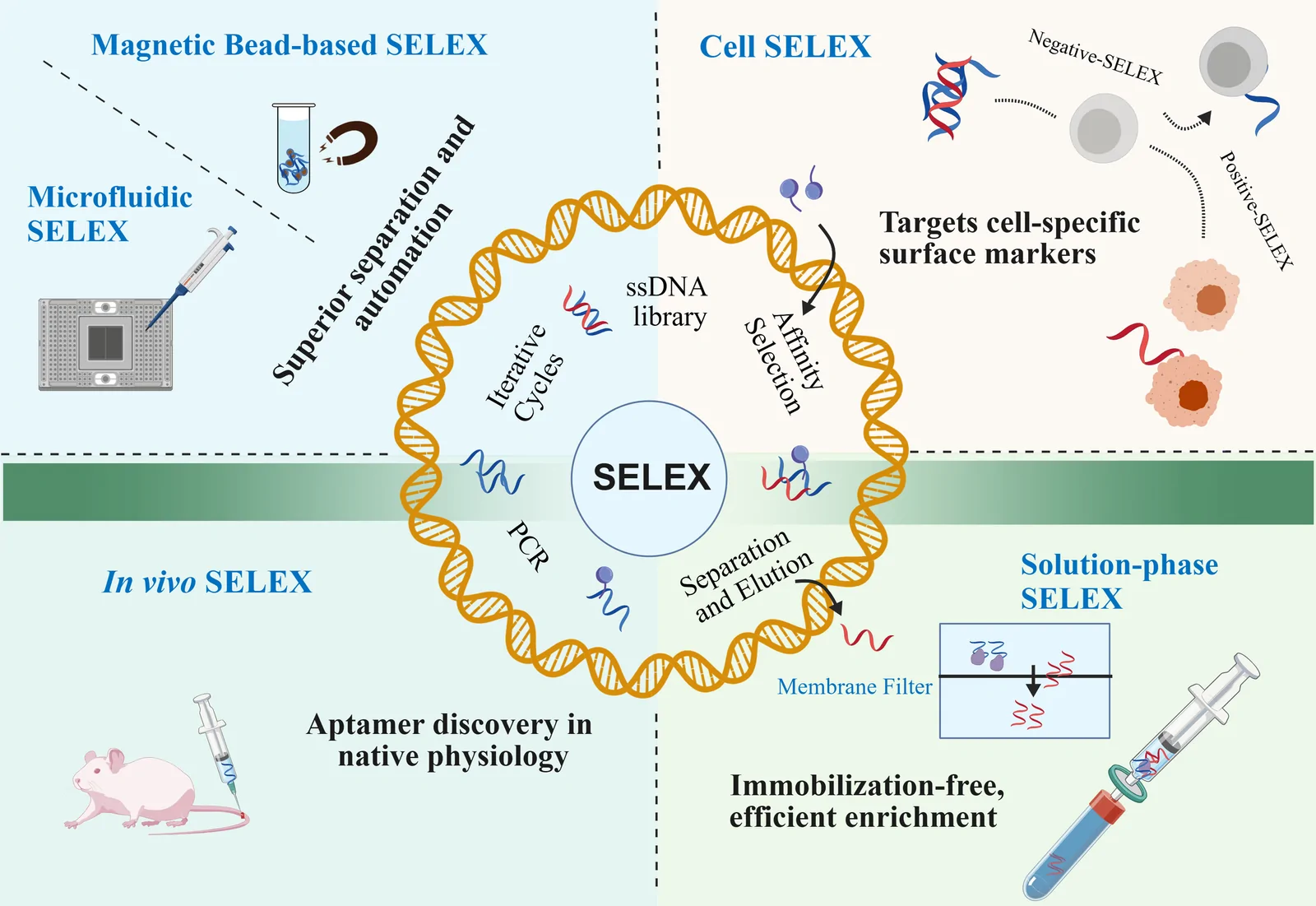
Schematic workflow of SELEX. The central schematic delineates the core iterative SELEX cycle (comprising ssDNA library construction, affinity selection, separation/elution, and PCR amplification), alongside several emerging variants such as Cell SELEX, *in vivo* SELEX, magnetic bead-based SELEX, and microfluidic SELEX. This figure was drawn with BioGDP and is an original work of the authors. BioGDP Agreement Number：GDP20268V0A4A. PCR: Polymerase chain reaction; SELEX: systematic evolution of ligands by exponential enrichment; ssDNA: single-stranded DNA.

### Evolutionary logic of selection technologies

Aptamer selection technologies have evolved through three overlapping phases. The first phase (1990-2010) established conventional SELEX against purified proteins, yet these methods proved ill-suited for EV targets owing to immobilization-induced conformational changes and high sample consumption. The second phase (2010-2020) introduced microfluidics and capillary electrophoresis, substantially reducing selection rounds while preserving native epitope conformations. The third phase (2020-present) is defined by automation, artificial intelligence, and functional selection - integrating robotic liquid handling, real-time monitoring, and machine learning to enable the fully automated selection of intact EVs without operator intervention, with functional activity as a primary selection criterion. Rather than a chronological catalog, we organize the discussion that follows around three core objectives that have driven this evolution: screening efficiency, target complexity, and aptamer functionality.

#### Improving screening efficiency

The past two decades have seen a paradigm shift from manual, time-intensive SELEX to automated, high-throughput platforms. Traditional SELEX suffers from a high number of selection rounds and long processing times^[8,9]^, as shown in Figure 3A. Integration of SELEX with microfluidics enables efficient aptamer separation, significantly shortening the selection time while minimizing the consumption of biological materials^[29,30]^, as shown in Figure 3B. Capillary electrophoresis SELEX eliminated target immobilization, a critical advantage for preserving native EV epitope conformations, and yielded high-affinity aptamers (Kd = 27.6 nM) in as few as five rounds^[28]^.

**Figure 3:**
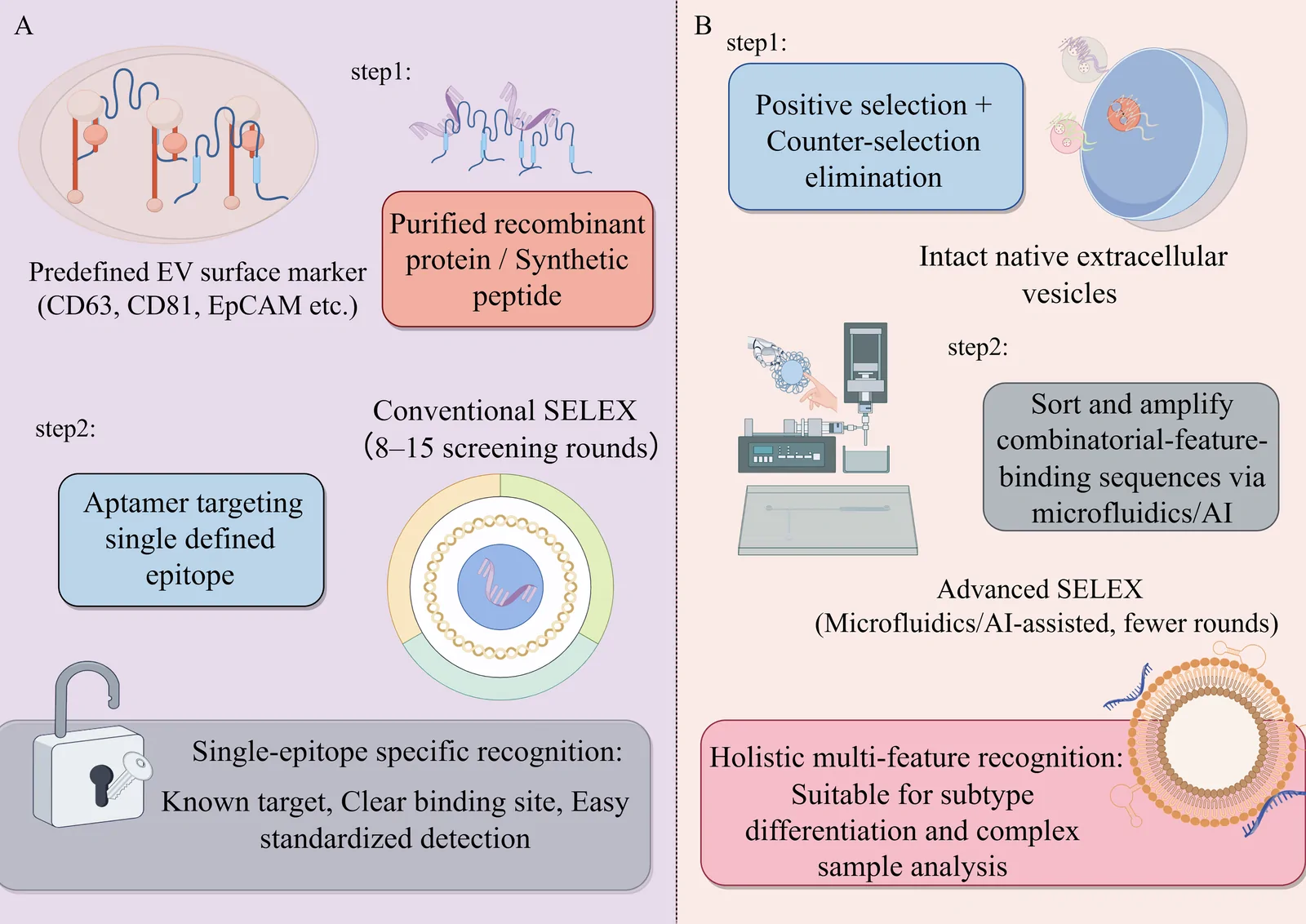
Comparative schematic workflow of SELEX methodologies for EVs. (A) Conventional SELEX strategy. This approach uses purified recombinant proteins or synthetic peptides as targets based on predefined EV surface markers (such as CD63, CD81, EpCAM). It requires 8-15 screening rounds and yields aptamers targeting a single defined epitope. This results in single-epitope-specific recognition, characterized by known targets, clear binding sites, and easy standardized detection; (B) Advanced SELEX strategy. This approach utilizes intact native EVs, incorporating positive selection and counter-selection elimination. It employs microfluidics/AI-assisted systems to sort and amplify combinatorial-feature-binding sequences, requiring fewer rounds. This method achieves holistic multi-feature recognition, making it suitable for subtype differentiation and complex sample analysis. This figure was drawn with Figdraw and is an original work of the authors. Figdraw ID: WYTPRcaf81. AI: Artificial intelligence; EpCAM: epithelial cell adhesion molecule; EV: extracellular vesicle; SELEX: systematic evolution of ligands by exponential enrichment.

Most recently, fully automated platforms integrating microfluidics, robotic liquid handling, real-time quantitative polymerase chain reaction (qPCR) monitoring, and machine learning have achieved the automated, hands-free selection of intact EVs within days^[30]^. These advances have transformed aptamer discovery from an artisanal craft into an engineering discipline. However, these platforms, alongside existing hybrid approaches that combine robotic handling with microfluidic modalities such as droplet microfluidics or acoustofluidics, still fall short of addressing three essential prerequisites for automated multi-analyte liquid biopsy applications in a single instrument: (1) supporting the processing of large sample volumes; (2) enabling the processing of more than one sample at a time; and (3) providing orthogonal extraction mechanisms as required^[31]^. Figure 3 presents a comparison of conventional versus advanced SELEX strategies for aptamer selection targeting EVs.

#### Expanding target complexity

The shift from soluble proteins to intact EVs represents a fundamental expansion in target complexity. Early efforts focused on individual EV surface markers such as epithelial cell adhesion molecule (EpCAM), mucin 1 (MUC1), and CD63^[21]^; however, such predefined targets cannot resolve EV subset heterogeneity. The breakthrough came with whole-EV SELEX, which treats EVs as complex molecular assemblies^[26]^. Cell-SELEX against cancer cells followed by counter-selection against normal cells enabled the discovery of aptamers recognizing unknown, disease-specific surface features^[32,33]^. EDGE-SELEX applied to intact exosomes, together with immobilization-free SELEX that avoids conformational artifacts^[26]^, has further pushed the boundary. Most recently, AI-driven SELEX design has optimized library complexity for multi-epitope recognition on EV surfaces^[33]^, while fully automated platforms now enable programmable, high-throughput selection against intact EVs^[29]^. Collectively, these advances have shifted the field from targeting purified molecules to confronting physiologically relevant, complex targets.

#### Enhancing aptamer functionality

Initially regarded as antibody substitutes for detection, aptamers have undergone a functional redefinition: selection criteria now extend beyond binding affinity to encompass biological activity^[34,35]^. Emerging strategies prioritize aptamers that not only bind with high affinity but block protein-protein interactions, mediate drug internalization, or activate immune signaling^[36,37]^. Examples include aptamers that block heat shock protein 70 (HSP70)-mediated immunosuppression^[38]^, as well as aptamers that recognize membrane protein targets on exosomes derived from highly metastatic colorectal cancer and directly suppress exosome-induced pro-metastatic activities, such as cell migration, invasion, and angiogenesis^[39]^. This functional turn shifts the paradigm from binding to intervention, positioning aptamers as therapeutic effectors rather than passive targeting ligands.

### Unresolved bottlenecks in aptamer selection for EV research

#### Despite these advances, several challenges persist

In some reported cases, functional selection may involve a trade-off with binding affinity^[34]^. For instance, a study using the DNA aptamer GS24 targeting the transferrin receptor revealed that this aptamer exists in two conformations; however, only one of them (A-2F) possesses biological activity. Engineering modifications, such as truncation or mutation, were employed to stabilize the active conformation and enhance its biological activity; nevertheless, the affinity of the truncated variant GS24min was approximately five times lower than that of the parental GS24^[40]^. However, this trade-off is not a universal rule; whether it occurs depends critically on whether the aptamer’s epitope overlaps with a functional site (e.g., a receptor-binding domain) or a non-functional structural region^[34]^. Several examples demonstrate that aptamers can maintain both high affinity and full biological activity when the epitope is appropriately positioned^[41]^.

The observed trade-off in EV applications is primarily based on a limited number of systems, such as HSP70^[38]^, and should therefore be considered a cautionary observation rather than an established limitation. Nevertheless, for EV applications where aptamers must simultaneously recognize surface epitopes with high specificity and exert downstream functions (e.g., inhibiting EV-mediated signaling or directing cargo internalization), balancing these two properties remains an important design consideration.

Second, the inherent heterogeneity of EVs, both across and within populations, severely compromises inter-laboratory reproducibility. EVs derived from the same cell type can vary significantly in size, surface marker density, and cargo composition depending on culture conditions, isolation methods, and storage protocols^[5]^. This batch-to-batch variability means that aptamers selected against one EV preparation may perform poorly against another, even from the same nominal source^[23,24]^. To address this, the field urgently needs standardized biological and physical reference materials to benchmark aptamer binding affinity and specificity across different laboratories and platforms. Promising candidates include recombinant EVs (rEVs) engineered to mimic native biochemical and biophysical properties for quantitative data normalization, nanoerythrosomes, and engineered vesicles or synthetic liposomes with predefined sizes, refractive indices, and marker/epitope abundances.

Third, concurrent target discovery and functional aptamer selection without prior knowledge of EV surface features remains a technical blind spot. Most current selection strategies either begin with a known target [e.g., CD63, programmed death-ligand 1 (PD-L1)]^[21]^ or use whole-EV selection to generate aptamers without immediately knowing their binding targets^[32]^.

However, when an aptamer selected against intact EVs elicits a desired functional effect, such as blocking EV uptake or neutralizing a pathological activity, identifying its cognate surface epitope becomes a major bottleneck. Reverse engineering the target from a functional aptamer is technically demanding and time-consuming, typically requiring immunoprecipitation, mass spectrometry, or clustered regularly interspaced short palindromic repeats (CRISPR)-based validation. Until this blind spot is resolved, the systematic discovery of novel EV surface biomarkers that are both therapeutically actionable and diagnostically useful will remain constrained.

## APTAMER-MEDIATED EV ISOLATION AND PURIFICATION

### Principles and key advantages of aptamer-based isolation

High-purity EV isolation is a prerequisite for downstream molecular analysis and functional studies. Conventional methods, such as ultracentrifugation, density gradient centrifugation, size-exclusion chromatography, and polymer precipitation, are hampered by low purity, long processing times, heavy equipment dependence, and an inability to resolve EV subtypes^[6,7,42]^. Aptamer-based affinity capture overcomes these limitations through two distinctive advantages.

First, aptamers offer high specificity for EV surface epitopes, including those with low abundance or post-translational modifications^[43,44]^. Second, and uniquely, aptamers enable mild, non-destructive elution via complementary strand displacement, preserving EV integrity and cargo for downstream multi-omics analyses^[45,46]^. This contrasts sharply with antibody-based capture, which requires harsh conditions (low pH, high salt, or organic solvents) that compromise vesicle structure.

### Evolution of technology platforms for aptamer-based EV isolation

#### Magnetic bead-based platforms

Magnetic separation is the most established aptamer-based platform for EV isolation. Biotin-labeled aptamers are immobilized on streptavidin-coated magnetic beads^[47,48]^, enabling rapid EV capture under an external magnetic field. At the optimal aptamer concentration of 1 μM, the capture efficiency reaches 80%, significantly higher than the 65% achieved by conventional antibody-functionalized Fe_3_O_4_ beads^[48]^. Single-target magnetic separation has since been applied to various disease-associated EVs^[49]^.

Multitarget magnetic separation further improves specificity and coverage^[50,51]^: dual-aptamer systems integrated with graphene field-effect transistors permit label-free detection of cancer-derived vesicles^[42]^, while triple-aptamer electrochemical approaches simultaneously recognize multiple EV surface epitopes^[52]^. Critically, complementary strand displacement and deoxyribonuclease I cleavage have been optimized for non-destructive elution, both of which preserve EV morphology and membrane protein integrity^[53,54]^.

#### Microfluidic chip platforms

Microfluidics integrates EV isolation into miniaturized chip formats, substantially reducing sample consumption and processing time^[55]^. Aptamer-based microfluidic devices enable target-specific EV capture with efficiencies of 10^7^-10^8^ particles/mL^[56]^, while select filtration-based platforms enable direct EV isolation from whole blood without pre-processing^[57]^. Magnetic microfluidics further enhances isolation efficiency by combining magnetic separation with on-chip flow control, completing isolation within 1 h^[58,59]^.

However, substantial challenges remain. Wide variability in chip geometry, flow rate, and surface chemistry across laboratories hinders cross-study comparison, and the lack of standardized reference samples for calibration impedes clinical translation.

#### Smart responsive materials

Smart responsive materials enable dynamic control over EV capture and release. Thermosensitive aptamer-conjugated materials achieve > 60% recovery rate through temperature switching^[21]^. Photoactivatable magnetic beads offer spatiotemporal precision^[60]^. DNA hydrogels incorporating CRISPR/CRISPR-associated protein 12a (Cas12a) respond to EV recognition through conformational changes, enabling signal-triggered release. Moreover, these hydrogels possess a high encapsulation capacity, with a maximum of 7.6 × 10^7^ exosomes per 50 μL of hydrogel, which facilitates efficient EV loading for downstream analysis^[61,62]^; in this system, EV binding induces a conformational change in the hydrogel, activating Cas12a’s trans-cleavage activity, which then releases a reporter or triggers cargo liberation. Molecularly imprinted polymers combined with aptamers can recognize glycosylation patterns, a capability beyond conventional aptamers^[63]^.

Although these platforms have expanded the toolbox from passive adsorption to active regulation, most remain confined to benchtop operation^[64]^; the lack of integrated automated liquid handling and external temperature/light control modules continues to impede their routine use.

#### Integrated multidimensional platforms

Integrated platforms that combine isolation with downstream analysis enable sample-to-answer workflows. The CLCtips platform, for instance, integrates aptamer-mediated capture and on-chip enzymatic digestion for trace EV proteomics, identifying over 500 proteins from minimal sample volumes^[65]^. Microfluidic chips coupled with mass spectrometry or next-generation sequencing permit multi-omics profiling from single isolation runs^[66-68]^, while proximity hybridization-mediated FRET (Fluorescence Resonance Energy Transfer) assays achieve high-specificity EV detection without separate isolation steps^[69]^.

Key bottlenecks for integrated platforms include multi-step compatibility (capture, lysis, digestion, analysis), the purity-sensitivity trade-off, sample throughput, and automation costs.

### Current bottlenecks for aptamer-based EV isolation platforms

Magnetic bead-based platforms offer a leading combination of maturity and ease of use, with consistently outstanding capture efficiency^[47-49]^. Microfluidic platforms deliver the best combination of speed and purity^[58]^, but microfluidic and acoustofluidic methods also exhibit limited selectivity and frequently yield impure isolated samples. Immunoaffinity-based approaches can enhance specificity through molecular recognition; however, their applicability to multiplex separation remains constrained^[70]^. Smart responsive materials excel in controllable release and reusability^[21]^, yet their dependence on external temperature or light control modules complicates workflow integration^[71,72]^. Integrated multidimensional platforms minimize sample handling by combining isolation with downstream analysis^[66]^, but the purity-sensitivity trade-off remains unresolved^[73,74]^. Collectively, no single platform meets all clinical requirements. Every method involves intrinsic compromises between recovery efficiency, purity, scalability, and the maintenance of vesicular integrity^[75-77]^. Moreover, inter-laboratory variability remains a significant impediment, as even slight discrepancies in isolation or handling procedures can markedly affect exosomal yield, molecular composition, and downstream biomarker measurements^[77]^. In addition to these trade-offs, isolation time has emerged as a critically important parameter, particularly for clinical applications where rapid turnaround is essential for timely diagnosis and for preserving the integrity of labile RNA and protein cargoes. Extended protocols not only reduce sample throughput but also increase the risk of degradation and batch-to-batch variation, hampering translation into routine practice. To clarify this key metric, Table 1 summarizes the isolation times of major aptamer-based EV isolation platforms.

**Table 1:** Time comparison of aptamer-based EV isolation platforms

**Technology platform**	**Carrier**	**Time distribution**	**Ref.**
Magnetic beads	mesoporous Fe_3_O_4_@Au nanoparticles conjugated with CD63 DNA aptamer	Capture incubation: 60 min; Elution: 20 min	[48]
Magnetic microfluidic	Tentacle-like magnetic microspheres conjugated with CD63 aptamer	EV enrichment: 20 min; Exonuclease-I-mediated release: 30 min	[58]
DNA-based hydrogel	RCA-synthesized ultralong DNA chains containing polyvalent CD63 aptamer	Capture incubation: 30 min; Hydrogel formation incubation 30 min	[61]
Smart responsive materials	Thermosensitive copolymer PNB grafted with CD63 aptamer	Capture incubation: 120 min; Complementary-sequence elution: 15 min	[21]

Performance metrics are compiled from various studies using different EV sources (cell culture, plasma, serum), isolation protocols, and purity assessment methods (particle count, protein ratio, or marker enrichment); therefore, direct cross-platform comparisons should be made with caution. EV: Extracellular vesicle; PNB: poly(N-isopropylacrylamide); RCA: rolling circle amplification.

## APPLICATION OF APTAMER-FUNCTIONALIZED EVS IN DRUG DELIVERY AND IMMUNOMODULATION

Aptamers offer high affinity, low immunogenicity, facile chemical modification, and scalable synthesis, making them ideal targeting moieties for EV functionalization^[78,79]^. This section examines two major application domains: targeted drug delivery and immunomodulation. Within drug delivery, we organize by cargo type, namely chemotherapeutics, nucleic acids, and proteins, while highlighting a critical bottleneck common to all: endosomal escape.

### Aptamer-mediated targeted drug delivery using EVs

The core strategy involves anchoring aptamers onto EV surfaces to enable specific recognition of diseased cells^[80,81]^. Three functional modules, including targeting (aptamer), carrier (EV), and cargo (therapeutic), can be combined in various configurations.

### Targeted delivery of chemotherapeutic agents

Aptamer-functionalized EVs are engineered to serve as a safe and effective targeted drug delivery system for specific anatomical sites, improving long-term persistence and boosting the safety and efficacy of chemotherapy treatments^[82]^. Two internalization routes have been exploited: receptor-mediated endocytosis and membrane fusion. Most strategies rely on clathrin- or caveolae-mediated endocytosis initiated by aptamer-receptor binding^[83,84]^. For example, the nucleolin-targeting AS1411 aptamer delivers doxorubicin via clathrin-mediated endocytosis^[82]^, and mesenchymal stem cell-derived EVs combine intrinsic homing with aptamer recognition for dual targeting^[85]^. A disulfide linker between the aptamer and EV enables glutathione-triggered drug release in the cytoplasm^[86,87]^, while raloxifene-conjugated systems use lysosomal targeting to induce both autophagy and apoptosis, a dual mechanism with the potential to circumvent chemoresistance^[88]^. Representative examples of receptor-mediated endocytosis pathways are summarized in Supplementary Table 2. A systematic schematic overview of all four major internalization routes (clathrin-mediated endocytosis, caveolin-mediated endocytosis, macropinocytosis, and direct membrane fusion) and their representative applications in aptamer-EV delivery systems is provided in Figure 4.

**Figure 4:**
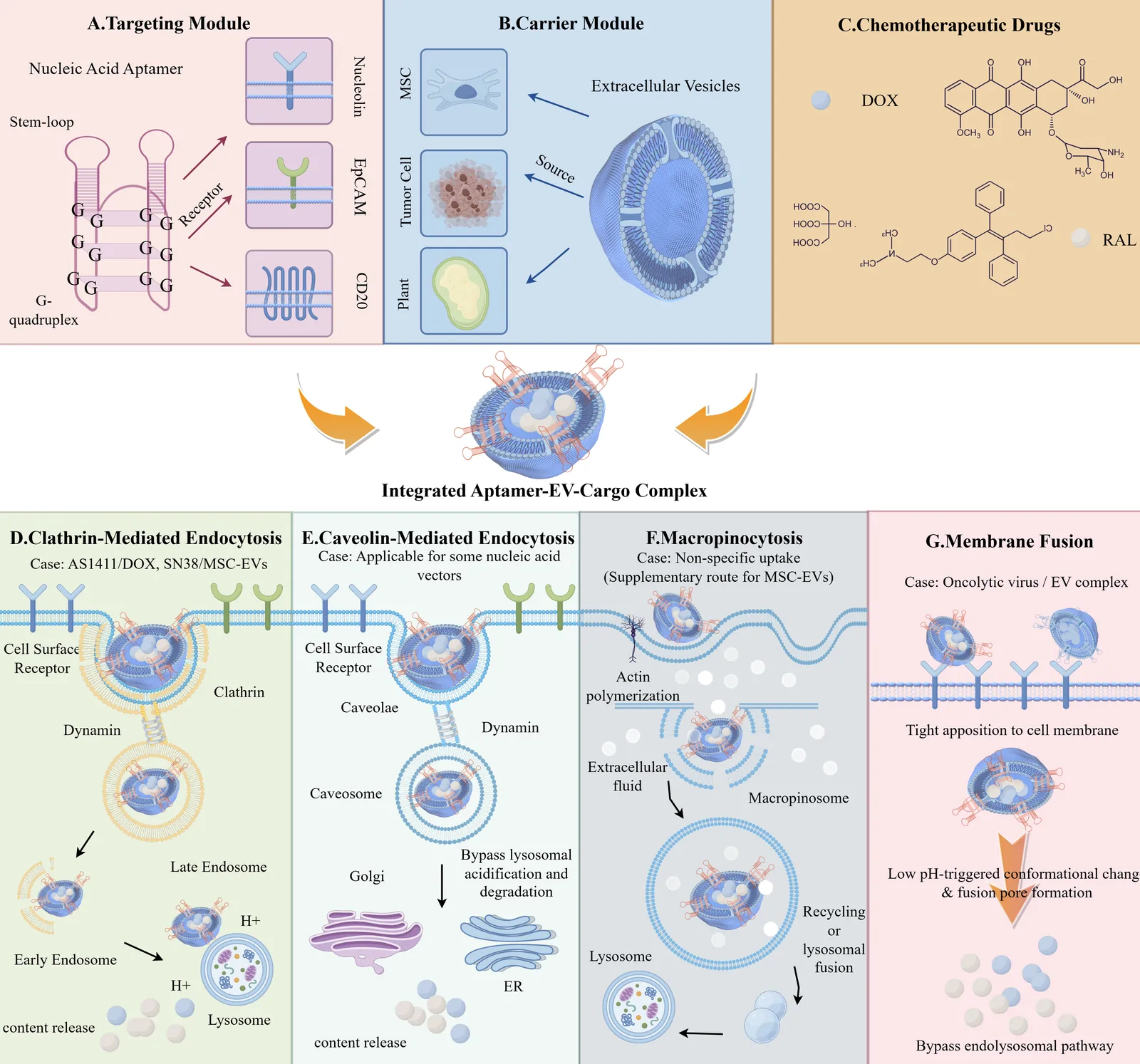
Modular design of aptamer-functionalized EV-based chemotherapeutic delivery systems. Upper schematic: This schematic illustrates the rational design of an integrated aptamer-EV-drug delivery system, which is assembled from three functional modules: (A) the targeting module, comprising nucleic acid aptamers with characteristic secondary structures (stem-loop, G-quadruplex) that bind to specific cell surface receptors (e.g., nucleolin, EpCAM, CD20); (B) the carrier module, consisting of EVs derived from multiple sources including MSCs, tumor cells, and plants; and (C) the chemotherapeutic drug module, represented by DOX and RAL. These three modules are conjugated to form the integrated Aptamer-EV-Cargo complex for targeted delivery. Lower subpanels (D-G): The lower portion delineates four distinct cellular internalization routes for the integrated complex, demonstrating versatile pathways for intracellular cargo release and potential avoidance of lysosomal degradation. (D) Clathrin-mediated endocytosis pathway, which is the primary route for AS1411/DOX and SN38/MSC-EVs internalization. (E) Caveolae-mediated endocytosis, suitable for nucleic acid vectors, which bypasses lysosomal acidification and degradation. (F) Macropinocytosis pathway, representing a non-specific uptake route that serves as a supplementary pathway for MSC-EVs. (G) Membrane fusion pathway, utilized by oncolytic viruses/EV complexes, involving tight apposition and low pH-triggered conformational changes to bypass the endolysosomal pathway. This figure was drawn with Figdraw and is an original work of the authors. Figdraw ID: TRSPAdd884. DOX: Doxorubicin; EpCAM: epithelial cell adhesion molecule; EV: extracellular vesicle; MSC: mesenchymal stem cell; RAL: raloxifene; SN38: irinotecan active metabolite SN38.

Despite these advances, all receptor-mediated pathways are constrained by inefficient endosomal escape^[89-91]^: only a minor fraction of internalized cargo reaches the cytosol^[92]^. This limitation is tolerable for potent chemotherapeutics but becomes critical for biologics. To demonstrate aptamer-mediated targeted docking, researchers developed a multifunctional exosome system, ExomiR-CVB3/DoxApt, comprising oncolytic coxsackievirus B3-derived exosomes^[93]^, a surface-conjugated AS1411 aptamer for nucleolin recognition, and encapsulated doxorubicin. In the acidic tumor microenvironment, aptamer-mediated nucleolin anchoring coincides with a sharp pH-dependent increase in drug release, resulting in localized drug deposition at the target cell surface^[94]^. This design provides mechanistic evidence for aptamer-assisted targeting: gel shift assays confirmed stable aptamer conjugation, and the differential release profile indicates preferential cargo unloading following aptamer-guided localization to the acidic tumor milieu. Nevertheless, these data offer indirect support for targeted docking; direct cell-binding or uptake studies are still required to fully validate the targeting advantage, and the potential off-target immunogenicity of the viral backbone must be carefully managed.

#### Targeted delivery of nucleic acid drugs

Target selection for aptamer-mediated EV delivery generally adheres to two criteria: (i) overexpressed surface biomarkers unique to pathological tissues [e.g., EpCAM, human epidermal growth factor receptor 2 (HER2), nucleolin, CD20]; and (ii) receptors capable of mediating robust internalization (e.g., transferrin receptor). Whole-cell SELEX offers an alternative route, enabling isolation of functional aptamers without prior target identification, provided they support intracellular uptake.

Nucleic acid therapeutics - small interfering RNA (siRNA), messenger RNA (mRNA), circular RNA (circRNA), and CRISPR-Cas9 - all require cytosolic delivery for activity. Aptamers serve a dual role, directing cell-specific uptake and, in certain designs, facilitating endosomal escape. For RNA interference, aptamer-displayed EVs have been validated for circRNA delivery to cancer cells^[95]^, and CL4 aptamer-modified EVs delivering long non-coding RNA (lncRNA) DARS-AS1 siRNA have been shown to reverse chemoresistance in triple-negative breast cancer^[96]^. For mRNA delivery, hepatocyte-targeted systems have been developed for familial hypercholesterolemia^[97]^; circular RNA and microRNA (miRNA) sponge delivery via aptamer-EV platforms has also been demonstrated^[98,99]^.

The most advanced application is CRISPR-Cas9 delivery. Elsharkasy *et al*. devised a modular strategy in which aptamers mediate ribonucleoprotein (RNP) loading into EVs, with ultraviolet (UV)-triggered release upon endocytosis^[100]^; Hegeman *et al*. further refined aptamer affinity to enhance delivery precision^[101]^; and Xu *et al*. engineered a reactive oxygen species (ROS)-responsive, aptamer-conjugated EV system for combined oncolytic virus OH2 and anti-PD-L1 therapy in prostate cancer^[102]^.

The endosomal escape bottleneck is most severe for these cargoes. Whereas a few siRNA copies per cell can achieve potent gene silencing, mRNA and CRISPR-Cas9 demand substantially higher cytosolic concentrations^[99,100]^. Current efforts to optimize escape through aptamer design - surface charge modulation or minor conformational adjustments - remain largely empirical and insufficient. Transformative progress will likely depend on equipping EVs with viral fusion machinery.

#### Targeted delivery of protein therapeutics

Protein therapeutics (molecular weight > 10 kDa, fragile tertiary structures) impose additional demands: protection during delivery^[103,104]^, controlled release, and evasion of ubiquitin-proteasome degradation. Aptamer-EV systems have addressed these challenges through three functional paradigms.

Functional antagonism: the aptamer itself acts as the effector. Gobbo *et al*. designed a peptide aptamer targeting HSP70 on tumor EVs, thereby blocking Toll-like receptor 2 (TLR2)/TLR4-mediated dendritic cell suppression^[38]^; Quintavalle *et al*. reported an aptamer against gremlin-1 that disrupts tumor-stroma crosstalk in breast cancer^[105]^.

Growth factor delivery: Luo *et al*. conjugated aptamers to bone marrow stromal cell EVs - directing them to hydroxyapatite or bone sialoprotein - while encapsulating bone morphogenetic protein 2 (BMP-2), thus shielding the growth factor from circulating proteases and preserving its bioactivity^[106]^.

Gene editor delivery: as described above, aptamer-EV systems for CRISPR-Cas9 RNP delivery represent the leading edge of this field^[101]^.

### Aptamer-mediated immunomodulation using EVs

Immunomodulation using aptamer-functionalized EVs spans three areas: cancer immunotherapy, immune cell engineering, and therapy monitoring.

#### Cancer immunotherapy

Three strategies targeting different nodes of the tumor-immune axis have emerged. The first strategy focuses on blocking immunosuppressive signals. Aptamers against HSP70 or PD-L1 on tumor EVs prevent these molecules from engaging immune receptors^[38,107]^, thereby restoring dendritic cell maturation and T cell activation. The second strategy involves delivering immunostimulatory agents. EpCAM aptamer-modified bacterial EVs serve as tumor vaccines, combining targeted delivery with natural adjuvant activity to enhance antitumor immunity^[108]^. The third strategy aims at eliminating suppressive EVs themselves. Aptamer-guided capture removes pro-inflammatory or immunosuppressive EV subsets, as demonstrated in allergic airway inflammation, and this approach holds promise for reshaping the tumor microenvironment^[109]^. Together, these three strategies illustrate the versatility of aptamer-EV platforms in reprogramming immune responses against cancer.

#### Immune cell engineering

Immune cell-derived EVs naturally carry major histocompatibility complex (MHC) molecules and co-stimulatory signals, making them attractive platforms for immunomodulation. However, their therapeutic utility is limited by poor targeting efficiency and heterogeneous production. Aptamer modification addresses the first limitation by enhancing the tumor-homing capacity of immune cell-derived EVs, enabling their accumulation at disease sites with higher precision^[109,110]^. To tackle the second, microfluidic strategies enable size-based separation of T cell receptor (TCR)-CD3 EV subsets, ensuring manufacturing consistency and reproducible therapeutic outcomes^[23]^. Beyond oncology^[111]^, this engineering approach has shown promise in inflammatory diseases: aptamer-functionalized EVs loaded on biomimetic periosteum can recognize nerve growth factor (NGF) released from injured neurons, delivering anti-inflammatory cytokines and neurotrophic factors to promote tissue repair^[112]^. Collectively, these strategies demonstrate that aptamer-mediated engineering can simultaneously improve targeting accuracy and manufacturing consistency of immune cell-derived EVs, addressing two key barriers to their clinical translation.

#### Immunotherapy monitoring

Real-time monitoring of immune checkpoint expression on circulating EVs is essential for evaluating patient responses to immunotherapy and guiding treatment decisions. Aptamers meet this need by enabling specific capture and high-sensitivity quantification of immune checkpoint molecules on circulating EVs^[113]^. For example, He *et al*. developed a wash-free, homogeneous assay based on an aptamer-cholesterol-anchored DNAzyme assembly, simplifying the workflow without sacrificing accuracy^[114]^. In another example, Cong *et al*. constructed a microfluidic device-based extracorporeal circulation system that combines CD63 aptamer-modified magnetic nanospheres for small extracellular vesicle (sEV) capture and a PD-L1-specific trigger aptamer for immune checkpoint recognition, coupled with hybridization chain reaction (HCR) for fluorescence signal amplification; this platform enables continuous *in vivo* monitoring of PD-L1^+^ sEVs during tumor progression, reflecting tumor burden and distinguishing early-stage from advanced disease, thus providing real-time feedback for immunotherapy management^[55]^. In addition, a range of signal transduction platforms have been adapted for aptamer-based EV detection, including SERS (Surface-Enhanced Raman Spectroscopy) probes^[115]^, quantum dot nanospheres integrated with machine learning^[116]^, ellipsometry, and thermophoretic assays^[117]^. All these platforms share a common three-step cascade - aptamer capture, signal amplification, and quantitative readout - with the principal differences residing in the signal transduction module and achievable sensitivity, entailing trade-offs among complexity, cost, and detection limits. A detailed comparison of these immune monitoring platforms is provided in Supplementary Table 3.

#### Tissue regeneration and other applications

Aptamer-EV systems have been applied to bone, nerve, and vascular regeneration. Luo *et al*. pioneered bone targeting by functionalizing bone marrow stromal cell (BMSC)-derived EVs with hydroxyapatite-binding aptamers and loading them with BMP-2^[106]^, while Su *et al*. achieved combined neural, vascular, and bone repair via NGF targeting and c-Jun N-terminal kinase 3 (JNK3) mitogen-activated protein kinase (MAPK) pathway activation^[112]^.

In neurodegenerative diseases, Ren *et al*. designed DNA aptamers against α-synuclein aggregates, enabling EV-mediated delivery of chaperones or autophagy inducers to attenuate pathology in Parkinson’s disease models^[118]^; Du *et al*. constructed multi-target engineered hybrid EVs bearing amyloid beta (Aβ)-specific aptamers for both oligomer neutralization and anti-inflammatory miRNA delivery^[119]^.

For anti-infection applications, Zhang *et al*. employed marine-derived EVs with bacterial-targeting aptamers for infected bone defect repair^[120]^, and Cui *et al*. developed a glycosylated extracellular vesicle-like receptor (termed GlycoEVLR) that enables rapid detection of viral antigens^[121]^.

Collectively, tissue regeneration and anti-infection applications constitute emerging frontiers where aptamer-EV systems offer distinct advantages, notably the capacity to target specific cell types or anatomical sites without requiring endosomal escape for therapeutic activity. Unlike intracellular drug delivery, where cytosolic cargo release is essential, tissue regeneration frequently benefits from sustained surface signaling or paracrine effects^[122]^, thereby mitigating the endosomal escape bottleneck. This distinction indicates that aptamer-EV technologies may achieve earlier clinical translation in regenerative medicine than in intracellular drug delivery.

### Key limitations of aptamer-engineered EVs for drug delivery and disease treatment

Despite the promising preclinical results discussed above, several critical limitations continue to impede the clinical translation of aptamer-engineered EVs. Three issues warrant emphasis. First, endosomal escape remains the dominant bottleneck for nucleic acid and protein cargoes^[89]^; even when aptamers mediate efficient cellular internalization, the vast majority of payloads are retained within endolysosomal compartments and fail to reach their cytosolic or nuclear targets. Second, the *in vivo* stability of aptamer-EV conjugates under physiological conditions - including exposure to serum nucleases, shear stress, and protein adsorption - is rarely systematically evaluated^[55]^, leaving their true pharmacokinetic profiles poorly defined. Third, manufacturing consistency^[23]^, spanning aptamer-EV conjugation efficiency and batch-to-batch EV homogeneity, remains unstandardized, posing significant obstacles to regulatory approval and scalable production. Future progress will depend on engineering active endosomal escape mechanisms (e.g., viral fusogens, pH-sensitive peptides), rigorous pharmacokinetic and pharmacodynamic characterization in relevant animal models, and developing scalable, Good Manufacturing Practice (GMP)-compliant production protocols^[116]^.

## REMAINING HURDLES AND EMERGING SOLUTIONS FOR APTAMER-EV TECHNOLOGIES

A schematic representation of the core challenges and future directions for aptamer-EV technologies is shown in Figure 5.

**Figure 5:**
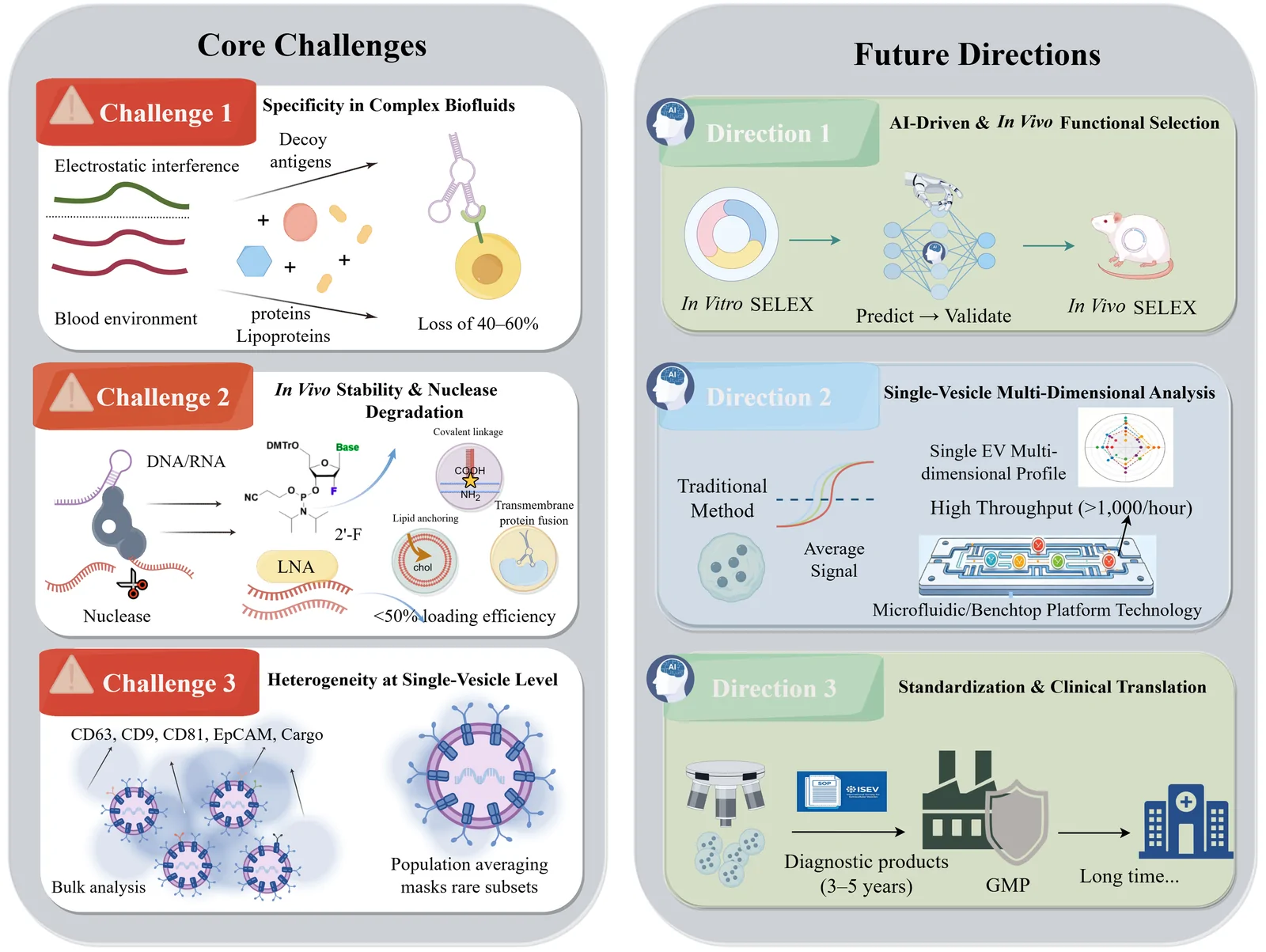
Core challenges and future directions in aptamer-EV technology. This schematic diagram summarizes the critical challenges and prospective future directions in EV-based research and therapeutics. This figure was drawn with Figdraw and is an original work of the authors. Figdraw ID: TRYAU8378a. AI: Artificial intelligence; EpCAM: epithelial cell adhesion molecule; EV: extracellular vesicle; GMP: Good Manufacturing Practice; ISEV: International Society for Extracellular Vesicles; LNA: locked nucleic acid; RNA: ribonucleic acid; SELEX: systematic evolution of ligands by exponential enrichment.

### Core challenges

Specificity in complex biological fluids: Biofluids contain abundant proteins, lipoproteins, and free nucleic acids that interfere with aptamer-EV binding. Non-specific adsorption, driven by electrostatic interactions between negatively charged aptamers and cationic proteins, can reduce effective aptamer concentration within 30 min in serum. Soluble antigens may act as decoys, consuming functional aptamers. Chemical modifications (e.g., 2’-O-methyl, locked nucleic acids) and inert carrier surfaces (e.g., PEGylated beads) partially mitigate these issues but may alter aptamer conformation^[7,123]^.

*In vivo* stability and nuclease degradation: The serum half-lives of unmodified DNA and RNA are 30-60 min and several seconds, respectively^[10]^. To enhance the serum stability of DNA-and RNA-based aptamers, various chemical modifications have been developed, such as 2’-aminopyrimidine, 2’-O-methylribonucleoside, and 2’-fluoropyrimidine. But excessive modification often reduces binding affinity. EVs themselves offer a solution as natural delivery vesicles: loading aptamers into EV lumens protects them from enzymatic degradation^[79]^. However, loading efficiency remains suboptimal and carrier-induced immunogenicity is a concern^[94]^.

Heterogeneity resolution at the single-vesicle level: Because EVs from the same parent cell can vary markedly in surface marker expression and cargo, bulk measurements inevitably average out subset heterogeneity. Current aptamer-based platforms operate at the population level^[31]^, masking functional differences between EV subpopulations^[30]^ that may hold distinct diagnostic or therapeutic significance. For instance, only a minor fraction of tumor-derived EVs may carry metastasis-promoting cargo, yet population-level analysis cannot resolve this subset from the predominantly benign background. Single-vesicle analysis, enabled by microfluidics coupled with single-molecule detection or high-resolution imaging^[46]^, remains at an early stage for aptamer-based platforms. Key technical hurdles include achieving sufficient signal amplification to detect low-abundance surface markers on individual vesicles (each EV carries only tens to hundreds of copies of a given protein) and maintaining high throughput^[59]^ while attaining single-vesicle resolution.

### Future directions

AI-driven and *in vivo* functional selection: Deep generative models and machine learning can predict aptamer-target interactions, optimize library design, and reduce selection cycles from 12-15 to 5-7^[14]^. Integrating multimodal data encompassing sequence, secondary/tertiary structure, chemical modifications, and target functionality will enable first-principles performance prediction, potentially reducing experimental screening to a validation step. Beyond the test tube, *in vivo* SELEX - direct selection in animal models followed by high-throughput sequencing, promises aptamers that withstand nuclease degradation and delivery barriers in native physiological environments. A key challenge remains: coupling *in vivo* selection with functional readouts (e.g., therapeutic efficacy rather than mere binding).

Single-vesicle multi-dimensional analysis: The convergence of microfluidics, single-molecule fluorescence, and aptamer-based recognition will enable simultaneous profiling of proteins, nucleic acids, and lipids on individual EVs^[66,67]^. This capability will permit deconvolution of EV subset heterogeneity and identification of rare, disease-secretory subpopulations, moving beyond population averages. However, single-vesicle analysis remains technically demanding: current methods offer limited throughput and require specialized instrumentation^[124]^. The near-term goal should be to develop benchtop-compatible platforms that can profile thousands of individual EVs within clinically relevant timeframes.

Standardization and clinical translation: Establishing standardized operating procedures and quality control systems for aptamer-EV technologies is an urgent priority^[125,126]^. Key parameters, aptamer sequence integrity, modification site occupancy, EV size distribution, and marker expression must be defined and benchmarked against reference materials such as those recently proposed by the International Society for EVs. Large-scale, multi-center prospective studies are needed to validate diagnostic, prognostic, and predictive performance^[127]^. Plant-derived EVs offer a scalable manufacturing platform^[128]^, but batch consistency and Good Manufacturing Practice compliance require further development.

## DECLARATIONS

### AI and AI-assisted tools statement

During the preparation of this manuscript, the AI tool ChatGPT (version GPT-4, released 2023-03-14) was used solely for language editing. The tool did not influence the study design, data collection, analysis, interpretation, or the scientific content of the work. All authors take full responsibility for the accuracy, integrity, and final content of the manuscript.

### Copyright

© The Author(s) 2026.
