# Supplementary material for: Aptamer-based technologies for extracellular vesicle analysis and engineering: from isolation to functional applications
Source: Extracell Vesicles Circ Nucl Acids. 2026 Sep 18;7(3):1496–516. doi: 10.20517/evcna.2026.106 (PMC13673466; doi:10.20517/evcna.2026.106)
Supplement: Supplementary file 1 [file evcna-7-3-1496-SupplementaryMaterials.pdf]

Supplementary Materials

Aptamer-based technologies for extracellular vesicle analysis and engineering: from isolation to functional applications

Xuefei Li[1,#], Youyu Zhang[2,#], Danping Zhang[1], Theerakamol Pengsakul[3], Ying Qiao[4], Jiang Zheng[1], Lixing Huang[1]

[1]State Key Laboratory of Mariculture Breeding, Fisheries College, Jimei University, Xiamen 361021, Fujian, China.
[2]Institute of Electromagnetics and Acoustics and Key Laboratory of Electromagnetic Wave Science and Detection Technology, Xiamen University, Xiamen 361005, Fujian, China.
[3]Health and Environmental Research Center (HERC), Faculty of Environmental Management, Prince of Songkla University, Hat Yai 90110, Thailand.
[4]Fourth Institute of Oceanography, Ministry of Natural Resources, Beihai 536000, Guangxi, China.
[#]These authors contributed equally to this work and share first authorship.

Correspondence to: Prof. Lixing Huang, Prof. Jiang Zheng, State Key Laboratory of Mariculture Breeding, Fisheries College, Jimei University, Xiamen 361021, Fujian, China. E-mail: lixinghuang@jmu.edu.cn; zhengjiang618@163.com

Supplementary Table 1. Comparison of aptamers and antibodies for EV-related applications

Comparison dimension	Antibody	Aptamer	References
Basic composition	Amino acids	Nucleotides (A, G, T/U, C)	[8,17]
Recognition principle	Antigen-antibody complex formation	Three-dimensional conformational complementarity	[11]
Preparation cost	High	Low	[9,17]
Batch-to-batch consistency	High variability between batches	Highly consistent across synthesis batches	[17]
Stability	Sensitive to temperature and pH	Very stable	[17]
Chemical modification	Difficult for site-specific modification	Flexible modification during or after synthesis	[12,17]
Immunogenicity	May induce immune response	Very low immunogenicity	[17]
Elution conditions	Harsh (low pH, high salt, organic solvents); damages EVs	Mild (complementary strand displacement); preserves EV integrity	[17]
Selection capability	Limited ability to apply negative selection pressure	Readily adjustable	[13]

Supplementary Table 2. Receptor-mediated endocytosis pathways in aptamer-EV drug delivery

Endocytosis type	Representative aptamer	Target	Cargo / EV source	Reference
Clathrin-mediated	AS1411	Nucleolin	Doxorubicin / engineered EVs	[82]
Receptor-mediated	Anti-colon cancer aptamer	Colon cancer surface antigen	SN38 / MSC-derived EVs	[85]
Lysosomal targeting	Raloxifene-specific aptamer	Estrogen receptor (ERα)	Raloxifene / aptamer-EV conjugate	[88]

Supplementary Table 3. Aptamer-based immune monitoring platforms for EV analysis

Platform	Capture element	Signal transduction mechanism	Target	Reference
Microfluidic device-based extracorporeal circulation	CD63 aptamer-modified magnetic nanospheres, IMNs	Hybridization chain reaction	PD-L1^{+} small extracellular vesicles, sEVs	[55]
DNAzyme assembly	Aptamer-cholesterol anchor	DNAzyme catalysis → fluorescence	PD-L1^{+} EVs	[114]
SERS probe	Aptamer-functionalized AuNPs	Surface-enhanced Raman scattering	PD-L1, CTLA-4	[115]
Quantum dot nanosphere	Aptamer-QD conjugate	Nanosphere amplification + ML	PD-L1	[116]
